# HIV-Associated Tuberculosis: Does the Iron-Regulatory Hormone Hepcidin Connect Anemia With Poor Prognosis?

**DOI:** 10.1093/infdis/jiv365

**Published:** 2015-07-01

**Authors:** Andrew E. Armitage, Ed Moran

**Affiliations:** 1 MRC Human Immunology Unit, MRC Weatherall Institute of Molecular Medicine, University of Oxford, John Radcliffe Hospital; 2 Department of Infectious Diseases and Tropical Medicine, Birmingham Heartlands Hospital, Heart of England NHS Foundation Trust, United Kingdom

**Keywords:** HIV-associated tuberculosis, hepcidin, anemia, iron, mortality

**(See the major article by Kerkhoff et al on pages 61–70.)**

Coinfection with *Mycobacterium tuberculosis* represents one of the major global health challenges associated with the human immunodeficiency virus type 1 (HIV-1) pandemic. HIV-associated tuberculosis is the leading cause of AIDS-related mortality, predominantly affecting resource-limited settings in sub-Saharan Africa [1]. Anemia is a frequent comorbidity of HIV infection, *M. tuberculosis* infection, and HIV–*M. tuberculosis* coinfection; in each case, anemia is predictive of mortality, independently of other well-established risk factors [2, 3]. The etiology of these infection-associated anemias is likely multifactorial, but anemia of inflammation (commonly referred to as anemia of chronic disease), which is associated with perturbations in iron status, may play an important role [4]. Sequestration of iron within macrophages accompanied by impaired iron absorption is commonly observed during inflammation, leading to functional iron deficiency and, if persistent, to iron-restricted erythropoiesis and anemia. Importantly, these phenotypes are also hallmarks of increased activity of the iron-regulatory hormone hepcidin.

Hepcidin is a liver-produced peptide that determines both systemic levels and anatomical compartmentalization of iron [5]. It is expressed in response to iron to maintain homeostasis but also as part of the acute phase inflammatory response, primarily via the interleukin 6/STAT3 pathway. Conversely, iron deficiency and periods of erythropoietic demand result in hepcidin suppression. Hepcidin activity causes degradation of the enterocyte- and macrophage-expressed iron exporter ferroportin, resulting in inhibition of dietary iron uptake, sequestration of recycled erythrocyte iron in macrophages, and reductions in serum iron [6]. Although anemia of inflammation may be caused in part by direct cytokine-mediated suppression of erythropoiesis, iron-restricted erythropoiesis mediated by hepcidin is also becoming well established as a key component of the pathogenic mechanism [7, 8].

Iron is a pathogenic determinant of many infectious conditions, not only because of its relationship with anemia, but also since invading pathogens typically require iron for effective replication [9]. The impact of iron status on infection pathogenesis likely differs according to the specific niches of the invading pathogens—whether they are extracellular, macrophage-tropic, hepatocytic, or erythrocytic [9]. While hepcidin-induced hypoferremia may protect against extracellular infections in mice, hepcidin activity and associated shifts in iron compartmentalization may differentially affect pathogens that use alternative niches, such as *Plasmodium* or *Salmonella* species [10–12]*.* The macrophage-tropic *M. tuberculosis* uses diverse means of scavenging host cell iron, including direct uptake of iron-loaded transferrin and heme, and by producing siderophores, such as mycobactins [13–15]. Similarly, HIV-1 replication can be enhanced by increased cellular iron [16]. Despite all of this, investigations of hepcidin's involvement in many human infectious conditions, including HIV–*M. tuberculosis* coinfection, remain limited.

In this issue of *The Journal of Infectious Diseases*, Kerkhoff et al present a detailed investigation of the relationships of hepcidin status with anemia, tuberculosis severity, and mortality risk in a well-characterized cohort of 232 HIV-infected adults from South Africa [17]. Participants were unselected, consecutively enrolled inpatients with newly diagnosed active *M. tuberculosis* coinfection or matched antiretroviral therapy–naive ambulatory outpatients with or without *M. tuberculosis* coinfection. The cohort included patients with pulmonary, extrapulmonary, and disseminated tuberculosis, allowing the most thorough observational evaluation to date of the behavior of hepcidin in this context.

While one might expect an acute-phase reactant such as hepcidin to rise in severe HIV–*M. tuberculosis* coinfection, hepcidin transcription may be simultaneously regulated by multiple inputs representing diverse physiological systems, so this should not simply be assumed [5]. For example, although hepcidin is upregulated during uncomplicated malaria, it may be suppressed during severe malarial anemia despite significant inflammation, presumably as a suppressive signal related to high erythropoietic demand dominating hepatic hepcidin regulation [18]. Likewise, hepcidin is suppressed during chronic hepatitis C virus infection, associating with the commonly observed hepatic iron loading [19]. Nevertheless, in the present study, the authors found that serum hepcidin concentrations increased with anemia severity and mycobacterial burden during HIV-associated tuberculosis.

The positive association between hepcidin concentration and anemia severity is consistent with hepcidin-mediated anemia of inflammation being a significant contributor to the etiology of HIV–*M. tuberculosis* coinfection–associated anemia. Correspondingly, a recent study of *M. tuberculosis*–infected patients from The Gambia (some of whom were HIV infected) found resolution of anemia following treatment in a significant proportion of individuals, accompanied by normalization of hepcidin levels; however, anemia was not universally corrected, likely owing to the presence of iron-deficiency anemia in the population, highlighting how anemia is often multifactorial in such settings [4].

In contrast to HIV–*M. tuberculosis* coinfection, Kerkhoff et al found no association of hepcidin with anemia severity in HIV-positive, *M. tuberculosis*–negative outpatients, suggesting alternative etiologies for anemia in these cases. Significant hepcidin upregulation has been described previously during the acute phase of HIV-1 infection and, similarly, during advanced HIV-1 infection [20, 21]. During the chronic asymptomatic phase, hepcidin levels may only be modestly raised or may appear relatively normal [20, 21], consistent with the present report. This suggests that the elevated hepcidin levels the authors observed in coinfection are primarily related to the mycobacterial infection, rather than to the viral infection. Accordingly, they reported that hepcidin levels were highest in the more severe cases of extrapulmonary and disseminated tuberculosis.

Kerkhoff et al generated multivariable Cox regression models, in which hepcidin was found to be an independent predictor of mortality in *M. tuberculosis*–positive, HIV-infected patients. For each 10-unit increase in hepcidin level (measured by the DRG hepcidin 25 enzyme-linked immunosorbent assay; note that absolute values returned by different hepcidin assays are currently nonequivalent [22]), an 11% increased risk of mortality was found. In contrast, although hemoglobin level was a significant predictor of mortality in univariate analysis, it was not predictive in the adjusted models, indicating that in this set of patients, hepcidin level was more important. A possible explanation is that, whereas there may be multiple causes of low hemoglobin level within this population, individuals with more severe inflammatory anemia, captured best by high hepcidin levels, have the worst prognosis. In other settings, hepcidin performs well as a single index capable of distinguishing inflammatory anemia from iron-deficiency anemia (in which the hepcidin level is low) [23].

So is hepcidin simply another acute-phase marker indicating which patients are sickest and accordingly have the worst prognosis, or could it be more intimately involved with the pathogenic processes occurring during HIV-associated tuberculosis? Are increased hepcidin concentrations a consequence or cause (or both) of worsening disease? The observational data in the present study cannot answer these questions directly, but they add significantly to previous clinical and experimental data in generating testable hypotheses regarding mechanisms.

The authors additionally observed highly significant associations between hepcidin and the acute-phase protein C-reactive protein, strongly linking hepcidin to the acute-phase response. Hepcidin upregulation is, therefore, highly likely a consequence of the inflammatory response to a developing infection. However, although C-reactive protein was predictive of mortality in univariate analysis, like hemoglobin it was not predictive in adjusted models, while hepcidin remained independently associated, consistent with hepcidin being more closely linked to the disease process.

Are there potential mechanisms through which hepcidin could be involved more directly in the pathogenesis of HIV–*M. tuberculosis* coinfection? On one hand, in vitro studies suggest hepcidin may have direct antimycobacterial properties (noting that the hepcidin concentrations tested were likely supraphysiological) [24, 25], so hepcidin upregulation in infected macrophages might represent a host response aimed at limiting infection. On the other hand, the better-established systemic function of hepcidin may simultaneously contribute to 2 processes relevant to clinical course: first, as described above, hepcidin may promote development of HIV–*M. tuberculosis* coinfection–associated anemia through serum iron restriction; and second, hepcidin activity enriches iron in the macrophage niche, potentially providing an iron source to favor mycobacterial (and, to an extent, viral) replication [9], while also potentially influencing macrophage immune effector functions [26]. Gene expression profiling suggests that *M. tuberculosis* experiences the macrophage phagosome as a relatively iron-poor environment, as several iron-acquisition genes, including siderophore genes, are upregulated [27]. Since iron is a crucial factor for *M. tuberculosis* growth in macrophages [13], increased macrophage iron retention during severe disease may, therefore, provide a source of iron to aid replication and further exacerbate disease in a positive feedback loop. Furthermore, hepcidin-mediated iron retention in lymphocytes may also enhance HIV-1 replication [16]. Additionally, recent studies report that altered iron indices including hepcidin predict subsequent diagnosis of active *M. tuberculosis* infection in HIV-infected individuals; while the times between iron status assessment and *M. tuberculosis* diagnosis were relatively short (in the order of months), the data suggest that perturbations in iron status related to hepcidin may precede development of active disease [21, 28, 29].

Should further basic science investigations provide mechanistic support for involvement of the hepcidin-iron axis in the pathogenic process of HIV-associated tuberculosis, a potential point of intervention may be revealed. Antagonists of hepcidin production or activity are currently under development, with anemia of inflammation a prime target [7]. Whether these would be effective as means of reducing the pool of accessible macrophage-based iron for *M. tuberculosis* and/or HIV replication while simultaneously alleviating HIV–*M. tuberculosis* coinfection–associated anemia is an interesting question worthy of further examination.
